# Supplementary material for: Prediction error signaling explains neuronal mismatch responses in the medial prefrontal cortex
Source: PLoS Biol. 2020 Dec 21;18(12):e3001019. doi: 10.1371/journal.pbio.3001019 (PMC7785337; doi:10.1371/journal.pbio.3001019)
Supplement: Attachment — Submitted filename: response to reviewers-0k.docx [file pbio.3001019.s006.docx]

Dear Dr. Gasque and Academic editor

First of all, we would like to thank the 4 reviewers for their constructive criticisms and yourself as the editor for offering us the opportunity to resubmit a revised version of our manuscript. It does not happen so very often but, occasionally, one has the privilege of receiving comments and reviews that are a pleasure to read.

We are very grateful and pleased with the reviewers’ comments on our manuscript for offering insightful observations and suggestions. Some of the comments are even raised by more than one reviewer which reveals their genuine constructive criticism. We honestly think they have helped us to improve this new and completely revised version of our manuscript. We think we have been able to successfully address all the reviewer’s comments and concerns, and that in doing so, the quality of the manuscript has notably improved.

The four reviews have been made with great care and detail, and from the reviewer’s comments it is patent that they have taken a significant time analyzing our results and interpretation thereof. We take that as a sign of acknowledgement of the potential interest of our work, and we sincerely appreciate their effort. Their comments are all pertinent, constructive and to the point, and in most cases concise and specific enough so as to facilitate the task of mending their reported concerns. We hope this new and much revised version is now acceptable. Below we provide extensive answers to their comments, clearly indicating the changes that implement each individual suggestion or request.

In the response to the reviewers, we explain and reply in detail to the specific comments one by one as listed by the reviewers and we have highlighted in blue our responses. We hope it is now acceptable for publication.

On behalf of all authors

Manuel S. Malmierca

----------------------------------------------------

**IMPORTANT - SUBMITTING YOUR REVISION**

Your revisions should address the specific points made by each reviewer. Having discussed these comments with the Academic Editor, we have formulated the following points which you should use as a guide during your revision:

1. We do not expect you to generate new data (experiments), but think that some of the analyses suggested by the reviewers are very pertinent.

We have done our best to address thoroughly every suggestion, but in some cases the characteristics of the data available prevented us to dig deeper, in particular in the cases of the laminar analysis and the characterization of the units by waveform analysis. Please see our response to Reviewer 3’ comment #3 for a detailed explanation.

On the other hand, we noted that several reviewers found troublesome the way in which we applied a baseline correction in order to remove the spontaneous activity from the evoked spike count and its derived metrics, such as the indices. The underlying reason was the long duration of the responses, that generated some amount of overlap among trials, and made very difficult to choose a proper time window with no evoked response, where we could accurately measure the spontaneous activity. This fact affected particularly the responses to the control conditions. It could be observed in Fig 1C from the previous submission, where the response to the controls during the “baseline window” was very similar to the “analysis window”; instead, the responses to deviant or standard conditions were not affected in this way. Since it was not possible to find a baseline time-window common to all conditions that properly allowed to extract the spontaneous activity in all cases, we have decided to repeat all analyses using the raw spike counts (i.e., without correcting for spontaneous discharge). An alternative would have been to record some “silence” time before each paradigm; unfortunately, we did not contemplate this possibility during the experimental design, so such data are unavailable.

This change in methodology reflects better the responses to the controls (e.g., Fig 2), which were underestimated in the previous manuscript. Likewise, the previous baseline correction produced some artifacts in the indices that are now avoided: iPE was overestimated (sometimes so much that the value of iPE surpassed that of the iMM, which should not be possible) and iRS was underestimated. Despite these miscalculations, the bottom line of the manuscript remains intact: the responses in the rat mPFC show strong iPE and weak iRS, especially when compared to the AC.

2. We agree with reviewer 3’s comment #3 that you should dig deeper into your laminar analysis and compare the different areas of mPFC rather than lumping them together. The ‘canonical circuit’ suggested for hierarchical predictive coding has clear predictions about where predictions error signals should be found (see for instance Keller and Mrsic-Flogel, 2018), as well as separation of feedback and feedforward. We think you have a very interesting dataset which you could potentially exploit more.

We also agree completely in the relevance of testing the postulates of the canonical circuit proposed for predictive processing. In that regard, we have checked our histological preparations trying to determine more precise locations for the units. Unfortunately, as we explain to Reviewer 3, due to the anatomy of the mPFC we have to rely on the electrolytic lesions, which are too large for this purpose. Instead, we have been able to assign a portion of the units to either superficial (layers II/III) or deep (layers V/VI,) locations, i.e., supragranular vs infragranular. The comparison of the responses in these two groups did not reach statistical significance. We discuss these results and its relationship with cortical implementations of predictive processing in L.198-208, L.355-357, L.385-387 and L.541-559.

In the current version we have looked more meticulously into the responses in each of the mPFC areas, adding information about LFPs and comparing them to the spiking responses. We also compare mPFC data with AC data from previous studies from our lab (Nieto-Diego et al., 2016; Parras et al., 2017).

3. A related point that reviewer 3 makes is about the lack of specificity. There is very little difference between the responses in the 4 subfields, and in fact in this experiment, we have no example of any area that does not present PE. Since you might have recorded from other areas in this experiment, we urge you to directly compare your results between the mPFC regions and these other regions, if only to make sure that this is not a kind of system-wide response.

While according to our previous data analysis, all mPFC areas showed a very similar and strong PE, we have now revealed several differences between them (partially thanks to the new analysis, which fixed the inadequate previous baseline correction). For instance, the strength of adaptation to repeated stimuli is different across areas (L.218-225). In the same line, M2 stands out from the other areas in being the only one where CTR and STD responses are significantly different (and thus having an iRS significantly different from zero, L.382, Fig. 3B), as well as in the shape and timing of its corresponding LFP (Fig. 4C,D).

Likewise, now we have performed a comparison between the mismatch responses found in the auditory system (data from Parras et al., 2017) and each of the mPFC areas, dedicating a whole section (L.290-350) and a figure (Fig. 6) to reveal the stark differences in PE and iRS between these regions.

4. You seem to be supporting the hierarchical predictive processing (HPC) model, but the argument in the discussion is based mainly on the amplification of response in mPFC relative to the auditory cortex (compared to data from Parras). However, the hierarchical nature of the HPC (a la Friston and others) is not about amplification of errors from lower to higher levels. It is about higher levels predicting lower levels prediction errors. This should be clarified (see Chao et al., 2018).

We apologize for the confusion in previous explanations. We have totally recast and rewritten the manuscript, so we hope that now this matter is accurate and clear.

5. Another argument for the hierarchical nature of the system is the faster repetition suppression in mPFC (lines 457 to 464). However, the final statement of this paragraph is perplexing: “Hence, the present data is consistent with and further support the hierarchical predictive model where higher areas should encode a regular contextual expectation faster and then send a prediction signal to lower areas to cease responsiveness to subsequent matching inputs”. It is confusing because if indeed mPFC quickly encodes the regularity and suppresses PE signal in lower levels, then why do you find lower levels waiting 5-6 stimuli before suppressing the response?

We apologize for this confusing explanation. Hopefully, our interpretation of this matter is now better articulated in L.341-351 and L.474-504.

6. Finally, two reviewers commented on the argument for attention independence. Their perspectives are a bit different, but both correctly note that without manipulation of attention in the experiment, one cannot argue for attention independence. This is not a central argument in the paper anyhow, and we would recommend that you shy away from discussing attention, and rather refer to the stimuli as being task-unrelated, irrelevant, etc, or at least to state simply that being anesthetized, the animals were not selectively attending the stimuli.

We fully understand the criticism and acknowledge the experiments presented here are not adequate to draw any conclusions about the role of attention. Therefore, we have removed all mentions to attention from the manuscript.

References:

Chao, Z.C., Takaura, K., Wang, L., Fujii, N., and Dehaene, S. (2018). Large-Scale Cortical Networks for Hierarchical Prediction and Prediction Error in the Primate Brain. Neuron 100, 1252-1266.e1253.

Keller, G.B., and Mrsic-Flogel, T.D. (2018). Predictive Processing: A Canonical Cortical Computation. Neuron 100, 424-435.

------------

*RESPONSE TO REVIEWERS*
*****************************************************
REVIEWS:

Reviewer #1: I have previously reviewed this paper at a different journal. I am attaching my comments from that review below here, for the benefit of the editor. The authors have done a commendable job in addressing most of my concerns. My one outstanding comment would be related to the writing. It is still unnecessarily confusing and chaotic in many places. Just to illustrate what I mean using a few lines from the abstract:

1.      Line 44. "Consistently" should probably read "consistent"
2.      Line 45. "High hierarchical areas" should probably read "areas high in the hierarchy" or similar.
3.      Line 46. "encode a contextual expectation FAST" - imprecise language. What does fast mean here? The encoding is fast? If so, relative to what? Faster than in other cortical areas? You can just remove the word "fast" from the sentence to improve accuracy of phrasing.
4.      Line 46. "send a prediction signal to lower areas to CEASE responsiveness" - combination of grammatical and logical problems. First, this should probably read "suppress" not "cease". Second, the point of the predictive suppression is to suppress responses to predictable stimuli to allow for strong responses to unexpected stimuli, not to "suppress responsiveness", which would imply that all responses are suppressed.
5.      Line 47. The sentence starting with "accordingly" I do not think I understand at all. Again, probably a combination of grammatical and logical problems. "The regularity of representation [...] was fast" what does that mean?

Similarly, throughout other parts of the manuscript. The discussion has much improved but is still quite chaotic and sometimes resembles a list of statements of fact, instead of a discussion.

If the authors can fix the writing, I would certainly recommend publication.

We would like to thank the reviewer for his/her comments. The whole manuscript (including the abstract) has been completely rewritten considering both the previous and current comments, and we have taken great care in a correct and clear writing. We hope that the reviewer will find a more cohesive and fluent narrative in the current version of the manuscript.

----------------------------------------------------------------------------------------------------
The comments I had in the previous review:

In this manuscript Casado-Roman and colleagues describe oddball responses in anterior cingulate, infra- and prelimbic cortex. They find very strong deviant responses consistent with a prediction error and compare these to previous work in auditory cortex. Overall the results are interesting, and if the authors can address the concerns detailed below, I would recommend publication.

1.      The manuscript would likely profit from being condensed. Many of the figures should be relegated to the supplements (e.g. 2,3 & 4). Similarly, in the description of the results, I would focus on the one main finding of the paper (strong oddball responses in fronto-medial cortex).
2.      The authors should more thoroughly discuss what the putative differences are to the Averbeck study (PMID: 30883292) and describe their findings more thoroughly. Statements of the form "However, one study in the PFC of the macaque with deep electrode recording failed to replicate the strong mismatch signals found in primates" are not only grammatically problematic, but also imprecise and do not do the cited work justice. E.g. what is a "deep electrode recording"? Should this read "found in rodents" - the Averbeck study is done in primates. Moreover, they do find deviant responses. Etc.
3.      Performing these experiments under anesthesia is somewhat problematic for an interpretation and should be addressed more carefully. It is clear that MMN can be measured in various behavioral states, but it is difficult to conclude based on this that response in higher order cortical areas are comparable under anesthesia and in the awake animal. The simplest way to address this would be to repeat the experiments in the awake rat, but maybe the authors have a different idea of how to best address this.


Minor

4.      The manuscript would profit from being proofread by a native speaker - there are numerous grammatical problems as well as typos.
5.      The cortical area the authors refer to as AGm is probably better described as M2 by modern nomencalature.
6.      Please include an example of raw recording trace.
7.      I would recommend formatting the manuscript in a form that is conducive to reading (e.g. keep figures and figure legends together).
8.      Abstract - "Beyond the auditory cortex, the prefrontal cortices integrate error signals to update prediction models." Please rephrase - unclear what is meant (what are "prediction models") and at best this is a theoretical concept not a fact.
9.      Abstract last sentence. Please remove or demonstrate how the authors' results relate to a "fundamental mechanism of hierarchical inference" (and explain what that means).

All of the reviewer’s comments from the previous review had been already fixed in the original submission. We never understood why the editor of that other journal rejected the manuscript without asking your opinion on our revision.

Regarding the requested example of a raw recording trace, which we were showing in Fig 1D in the previous version, since there is a conflict with the comments from other reviewers, it has been simplified and now it is shown as part of the schematic in Fig 1A.

Reviewer #2: The manuscript entitled "Prediction errors explain mismatch signals of neurons in the medial prefrontal cortex" by Casado-Roman and colleagues continues an important research theme of this lab to the medial prefrontal cortex, namely the detailed analysis of predictive processing in the auditory system. The results themselves are novel and exceptionally salient, the manuscript is well written and addresses most of the questions that came to mind while reading the results. My main concern lies with some of the statistical analyses, and while I think these need to be addressed, I don't think they would substantially alter the results or their interpretation.

We would like to thank the reviewer for his/her supportive comments and constructive criticism.

As mentioned above, my only significant confusion about the results lies in the statistical analysis in particular the statistics underlying the iRS evaluation. In several figures it appears that the standard responses are significantly smaller than the controls, e.g.
- grand average in Figure 1C (and others), where SEMs are indicated, which should translate to significant differences,

Former Fig. 1C was intended to explain the several time windows used. That figure was misleading, because it showed the responses before baseline correction. However, such correction was applied to the analyses performed afterwards, so the data values presented in the paper did not seem to match closely the data shown in the figure. Furthermore, the selected baseline window was problematic, since it captured strong responses in the control conditions, leading to an underestimation of iRS and an overestimation of iPE (please see the response to the editors for a more detailed explanation). Given the difficulty to select an appropriate baseline correction window, we have repeated the analyses without correcting for spontaneous activity. Now the iRS is significantly greater than zero in the mPFC as a whole (although quite small in size, median iRS = 0.06; L.305), as well as in M2 (when considering each area separately; Fig 3B).

- individual responses in Fig.4, comparing the standards to the embedded standards in the controls, there seems to be a significant difference for most areas (I assume that every dot corresponds to a single standard response, which could be described a bit more clearly).
Clearly, these would lead to smaller iRS values in comparison to the iPE, but still significant. In contrast all your tables and texts indicate that there are no significant differences between the controls and the standards in these conditions (at least when distributed across areas). Of course it could be the case that the statistical power is too low for individual areas (given the comparably low number of multiunits), but it does not seem so from the plots. I would ask the authors to quantify this, also on the across-area level and potentially correct their conclusion about no iRS effect at all, to a small, but significant effect.

Please see the response to the previous comment.

We hope the new explanations are clearer and more accurate for the plots shown in the former Fig.4 (current Fig. 3D) in L.211-215.

Also, former Fig.4 is probably not the best one to compare the responses to control and standards. Instead, we invite the reviewer to inspect the current Fig. 3B, where the responses to the different conditions are shown side by side.

Minor points;
Fig. 1D: The representation of the inset is a bit midleading, since the response seems to come long-time after the stimulus (this clarifies later with the long response latencies in mPFC). Could you indicate the time-scale for this inset, and maybe also show an estimate of response latency already.

Former Fig. 1D was an attempt to show an actual recording with multiunit activity preferentially in response to deviant tones, since reviewer 1 requested a raw recording in a previous submission. We have simplified the plot, in an attempt to please both reviewers, and more specifically, to better depict the data acquisition process. This new figure shows a raw recording integrated in a schematic of the experimental approach (Fig 1A).

Fig.S1: I think it would be nice to integrate Fig.S1 into Fig.1 to immediately clarify the relative spatial location of these areas.

We have reorganized all figures, and included the relevant information from the previous Supplementary Figures into regular Figures. This information from Fig. S1 now appears in Figs 1A and 2. We are very pleased to have eliminated the supplementary figures and include these data in the main manuscript figures as we believe it is more comprehensive.

L81: Wouldn't it be more accurate to say: "The predictability of stimuli reduces the PE signals, which has previously been described as repetitions suppression (RS) on a phenomenological level." If so, this would need some more changes, e.g. in lines 85/6.

This sentence has been removed in the current rewrite.

L306 What does this sentence mean?: Within these conditions, two tones were never repeated and did not undergo the same influence of the repetitive effect as standard events

We agree that this sentence is unclear. Here we meant to explain that during the deviant, many-standards and cascade conditions, the same tone was never repeated in two adjacent trials (opposite to what occurred during the standard condition).

The section has been rewritten and this unclear sentence removed (L.211-233). We hope now is more certain.

L.329: why is the rate of RS described as comparable, if there is a two-fold difference between M2 and PL? Form the plots, ACC should have the slowest decrease, which should result in a smaller absolute exponent b.

We agree with the reviewer that “comparable” is not the best adjective in this case. Instead, we now mention that “The repetition suppression manifested very fast and robustly in all mPFC fields” (L.227)

L335: I find this characterization a bit 'out of context': for the controls, not only the 'standards' but every other stimulus is repeated equally often. So, I think it would be more accurate to also analyze the activation of these stimuli in addition (where you would likely also find that they are equally suppressed, probably because they are not so 'statistically unlikely' (within their respective sequences), compared to the deviants (which you indicated with the words 'when acoustic input matches the current prediction', but this could be more explicit).

We have rewritten the description of the conditions and recording procedures, so they appear clearer to the readers (e.g. Methods, L.675-705). In short, many of the tones from the control sequences were also tested in the deviant condition (as many as the recording conditions allowed, a total of 384 tones from 83 units), so they are already included in this analysis. Also, in our opinion the strength of this analysis lies in the comparison of the time course of the responses when the same tone is presented in the different conditions; showing the responses to control tones without their corresponding deviant or standard would lack the necessary context.

Fig. 4: A and B labels missing.

Thank you for noticing these missing labels. This Figure has been replaced in the current version.

Reviewer #3: Review PBIOLOGY-D-20-00821R1

Summary
Casado-Román and colleagues present a very interesting study about sensory prediction error signals in the Prefrontal Cortex of anaesthetized rats. The goal of this study is to identify the involvement of prefrontal cortices in the generation of prediction error signals and Mismatch Negativity. By presenting different sequences of acoustic stimuli and multi-unit recordings they isolate very strong prediction error (PE) signals in 4 different fields of the medial prefrontal cortex (PrL, IL, ACC and M2). This is certainly by far the clearest and strongest demonstration of a PE signal on the level of multi-unit activity and deserves a lot of credit. This observation is an important confirmation of the model of hierarchical predictive processing in the brain. Therefore, I consider this study an important contribution to the field. However, I think the manuscript in its current form remains too much on a phenomenological level, describing a strong effect with the classical techniques
of extracellular electrophysiology. Extending the analysis and probably performing additional experiments may help to provide a deeper insight into the mechanism underlying this very important observation

We would like to thank the reviewer for his/her supportive comments and constructive criticism

Major comments
#1 Prefrontal Cortex: I think the authors have to be a bit more careful with their comparison of rodent prefrontal cortex (PFC) and primate PFC. The authors very clearly state in the introduction that previous studies investigating predictive activity in the primate PFC (e.g. Camalier et al. 2019, roughly area 46/8Ad) lacked some critical controls (e.g. see Introduction, line 103-108). The current study is supposed to fill this gap by measuring predictive activity in the rat mPFC with the necessary control conditions to isolate the predictive component (Introduction line 114-116). However, I would argue, that the primate PFC is fundamentally different from the rodent PFC, in particular areas like 46/8 which may have no homologue in rodents at all (e.g. see Wise, 2008: Forward frontal fields: phylogeny and fundamental function or Roberts and Clarke, 2019: Why we need nonhuman primates to study the role of ventromedial prefrontal cortex in the regulation of threat- and
reward-elicited responses). Actually, the rodent infra-, prelimbic and cingulate regions are typically compared to primate areas 25, 32 and 24 and not 46 and 8 (where Camalier et al 2019 recorded). I suggest that the authors make it very clear that they are not studying a direct homologue of primate PFC and that the comparison and translation of their results to primate PFC may be difficult and very limited.

We apologize for giving the impression that the rat PFC and the primate PFC are directly comparable, when there are striking differences as the reviewer correctly indicates.

Now we explicitly acknowledge the differences between species (e.g., L.405-407), and have taken care to avoid similar confusions throughout the manuscript.

#2 Attention: Throughout the manuscript there are repeated references to attention and the need to study "[…] whether predictive activity independent from attention emerges at the cellular level in the PFC" (Introduction, Line 98-99), see also in the Abstract, line 36-38: "[…] unknown whether prediction error signals are encoded at the neuronal level independently from attention or experience-dependent expectations." and Discussion line 427. I think it is misleading to say that the presented study allows to demonstrate the attention-independency of single/multi-neuron predictive activity. This question cannot be answered with the experiments under anesthesia as presented in the current manuscript and this is acknowledged by authors in the discussion as well (line 393 - 394). I think, this statement should be removed from the Abstract and Introduction or formulated more specific. The use of Urethane as anesthetic is not sufficient to support this view. Only experiments with
control over the attentional state of the subject could do so.

The reviewer is right that the experiments presented here are not adequate to draw any conclusions about the role of attention in the responses of the PFC, and apologize for being overly speculative in this matter. Subsequently, all references to attention have been removed from the manuscript.

#3 Lack of specificity: Across all areas the authors demonstrate a very strong prediction error signal which is interesting. However, this is a bit surprising as well because the different PFC areas investigate are quite diverse (IL, PrL, ACC and M2). If this strong PE signal indeed is present in all 4 areas and not a result of the unspecific nature of multi-unit recordings, it points to another brain area computing the prediction error and broadcasting it to the PFC areas studied here. The other problem in this study is the that we don't learn anything about the neuronal mechanism behind the observed phenomenon. Again, an increase in specificity may help here, e.g. distinguishing between cell types, layers in individual areas and sub-areas.  The authors analyze the layer-specific activity coarsely (Results, line 234-240), however they do so for all areas together.
A better insight into the underlying mechanism may be achieved partially by a more detailed analysis of the existing data (e.g. spike sorting and waveform analysis) or by additional experiments increasing the sample size per layer and area, targeting specific cell-types or inactivating individual (PFC) areas in order to test if the prediction error originates in one of these areas or is indeed almost ubiquitous in the PFC. In the discussion, the authors point towards these approaches (line 486-491) and they should seriously consider to pursue them.

While the responses across the different mPFC areas show many similitudes, it is worth noting that there are some differences. For instance, the strength of suppression of the response to repeated stimuli is different across areas (L.218-225). In the same line, M2 stands out from the other areas in being the only one where CTR and STD responses are significantly different (and thus having an iRS significantly different from zero, L.382, Fig. 3B), as well as in the shape and timing of its corresponding LFP (Fig. 4C,D).

The anatomical location of the PFC made it difficult to perform a more detailed analysis of the layers. In other cortical structures, such as the AC, it is possible to position the electrode perpendicular to the brain surface (and thus the layers), and then obtain precise electrode depth measurement by reading the microdrive displacement. In the PCF, on the other hand, the electrode enters the brain more or less parallel to the layers (e.g. Fig 1A), so the estimation of the recording location relies on the electrolytic lesions. Since such lesions extend tens of microns (we show some examples in Fig 2A), they lack the necessary accuracy to assign the recording location to any layer with precision. Despite this limitation, we could assign a portion of the recordings to superficial or supragranular (layers II/III) versus deep or infragranular (layers V/VI) locations, which is what we tried here, but we did not find any significant changes between both groups (L.198-208).

We agree with the reviewer that distinguishing between cell types would be a valuable addition in order to dig deeper into the mechanisms of deviance detection in the PFC. One way of doing that would be to perform a spike sorting analysis to isolate single units, and then measure spike waveform parameters that would allow a distinction between putative excitatory and inhibitory neurons (e.g., Pérez-González et al., 2020). Unfortunately, we found difficulties to obtain single unit recordings in the PFC, therefore the quality of the data only allows us to present them as multiunits, so we regret to say that it is not possible to extract that kind of information from the current recordings.

These methodological limitations are acknowledged in the manuscript (L.540-550)

#4 If the authors indeed measured a single-neuron correlate of MMN, as they suggest, it should be visible in the local field potentials (LFP) as well or at least EEG. It should be pretty straight forward to extract and analyze the LFPs data and check if there is a signature that resembles the human or rat MMN.

We thank the reviewer for this comment. This was an excellent observation and therefore, we have analyzed the LFPs. Indeed, we found strong LFP signals in response to the deviants that correlated with the timing of the spiking responses, while the LFP in response to the standards or the controls was very weak (L.260-276 and Fig.4C-D). Based on this analysis, now we also argue that our recordings are compatible with the frontal MMN source (L.418-428).

#5: Methods, line 647-649: units had to exhibit a significant increase in firing in at least one of the stimulus conditions (deviant, standard, cascade or many standards, compared to baseline) in order to be included in the analysis. This selection may distort the dataset significantly towards units exhibiting some kind of oddball/PE effect. Therefore, it is important to know if these units are accounted for in table 1 or not (as I assume). If they are not accounted for in table 1, the authors should provide the number of units that were excluded on the basis of this criterium.

Because of the problems generated by the baseline time windows, we do not compare the responses to the baseline anymore (please see the response to the editors for the reasoning behind this decision).

Minor comments
General: Throughout the manuscript, I find the terminology of at "target" tone misleading (e.g. Methods, line 610 and other places). Typically, a target stimulus is used in the context of a behavioral paradigm (e.g. a stimulus to be detected by the subject). However, in the current study no behavior was investigated.

We have replaced “target tone” with “tone of interest” to avoid this misperception.

Abstract, line 35-36: "cortex is postulated to integrate multiple prediction error signals and generate top-down predictions of sensory inputs." The authors assume different prediction error signal generators/sources. But how is this assumption justified?

This sentence has been removed during the revision, but we discuss the existence of multiple sources of prediction error both at cortical (L.79-91) and at subcortical levels (L.506-538)

Introduction, line 80-81: "PE signals are suppressed through a process of repetition suppression (RS)". Is there a reference for this statement?

We have removed this sentence in the current version of the manuscript, but references about repetition suppression as the reduction of response during and oddball paradigm can be found in L.99

Introduction, line 92-93: "ECoG reflects the population network dynamics because it records high gamma activity (~80-150 Hz) that reflects multiunit but also synaptic activity." The relationship of high gamma and multi-unit activity in ECoG signals is actually controversially discussed. Apart from that, I don't understand the function of this statement in the context of localizing potential generators of MMN in the human brain and the limited spatial specificity of intra-surgical ECoG mapping.

We agree that this statement was controversial and out of place, so we have removed it.

Figure 1C: The "Baseline window" (dotted lines) seems to overlap with the preceding "Analysis Window" (dashed lines), including still a period of increased activity from the preceding stimulus (grey lines). How ill this affect the analysis?

The long latency and duration of the responses found in the PFC made it difficult to choose a proper time window to measure the baseline activity. Typically, we would have chosen for that purpose a time window just before the stimulus onset, but found that in many cases the response to the previous stimulus would enter that time window. Based on the long latencies found in our recordings, where the bulk of the response occurs in the range of ~100-600 ms, we considered it was safer to delay both the baseline and the analysis time windows (please see Fig 1C in the previous submission).

However, this approach was still troublesome, as several reviewers have commented. The main problem is that the 0-50 ms baseline window, while it was rather appropriate for the standard and deviant conditions, it was in a region with strong response to the previous stimulus in the control conditions, causing serious artifacts in the measurements. Since there is not a single time window that can be used to reliable measure spontaneous activity for all conditions, we have decided to repeat the analyses without applying a baseline correction (for a more detailed explanation, please see the response to the editors).

Figure 1D: I am not sure I understand what the function of this panel is. It makes sense to show some examples of raw data or individual PSTHs/example units (as in supplementary figure S2). But just a trace of high-pass filtered multi-unit activity is not very informative.

Former Fig. 1D was an attempt to show an actual recording with multiunit activity preferentially in response to deviant tones. Reviewer 1 requested a raw recording in a previous submission. As a compromise, we have simplified the plot, and now show a raw recording integrated in a schematic of the experimental approach (Fig 1A)

Table 1, Results line 135-138, Methods: If I understand it correctly, for individual units, the authors analyzed all stimuli that evoked a significant response. However, statistically, it seems that all these different stimuli tested even in the same unit are treated as independent, increasing the statistically power massively. But this is not justified when they just come from a small group of neurons, tested with different stimuli.

We decided to process our multiunit responses in this manner following the methodology of previously published studies (namely Parras et al., 2017; also Parras et al., 2020 and Valdés-Baizabal et al, 2020), in order to allow comparisons between the mPFC and data recorded along the auditory system. In the current study, we have tried to replicate the methods of those works in the auditory system, including their statistical processing.

Figure 3 legend, line 267: The authors state "[…] mPFC neurons show a long and robust response to deviant events decaying with the next standard tone." (see also Results, line 251-252). This statement is only true when the all units and tested frequencies are lumped together. If one looks at Figure 3C it is obvious that each individual neuron has a peak of activity when Deviant stimuli are presented and that this peak ranges from approx. 100 ms to 700 ms post-stimulus.

The reviewer is right. Such description only applies to the “grand average” of the response, so we have removed it. For each individual unit and frequency, there is a peak with variable latency. During the rewrite we found that this figure was confusing and not very informative, so we decided to remove it.

The average spike-density functions for each area can be found now in Fig. 4B, and examples of individual units are shown in Fig 2D. We have also included an analysis of the response peak latency (L. 236-250).

Results, line 287-289: "In the mPFC population, latencies of maximum firing rates to deviant events varied notably among frequencies and were uniformly distributed within each field (Fig 3C)." In Figure 3C top row, the distribution of peak latencies seems to be more sigmoid than uniform (at least for M2 and ACC).

As mentioned in the previous response, we found that Fig. 3C was misleading, so we removed it as well as the related explanation.

Results, line 342ff and Figure S3: Probably the "inverse" experiment could be interesting as well. Instead of "Deviant-alone" condition (where the deviant "violates" the preceding silence") stimulus omission could be tested. This is one of characteristic features of MMN that even a "no stimulus" event violates are rules and gives rise an MMN wave (e.g. see Yabe et al. 1997).

This is a very insightful comment. The omission sequences are of great interest to our group. We have already presented some data recorded in the AC and IC (ARO Meeting 2020, abstract 852), and we are currently working to submit it in a separate paper.

Methods, line 545-546: How was the multi-unit activity filtered (type of filter, order, etc.)

The multiunit activity was band-pass filtered between 500 and 4500 Hz using a second order Butterworth filter (L.616).

Methods, line 550-557: How were the electrolytic lesions performed (current, duration, configuration etc.)?

The lesions were made by a single pulse of 5 µA during 5 seconds (L.708)

Methods, line 535: the stereotaxic coordinates have a large variation, approx. 2mm into both the rostro-caudal and the medio-lateral direction. Why was that so? This may significantly reduce the comparability of recording sites between animals and areas as different subareas may have been targeted.

We strived in obtaining reliable recordings from the mPFC, to the point that we had to settle with multiunit recordings when we were expecting to obtain single unit recordings. Since mPFC units do not respond clearly to pure tones, we encountered some difficulties in finding them, and many times we had to try multiple penetrations, positioning the electrode in different coordinates, until we could obtain a reliable signal. However, we performed the electrolytic lesions to confirm the recording locations and assign them to the proper mPFC area, although we lack the precision to locate them in smaller subareas.

Methods, line 563: what type of speaker has been used for the auditory stimulation? And what is the definition of a "flat spectrum"? How many dB deviation was accepted?

A custom-made close-field speaker was used in these experiments. By “flat spectrum” we mean that, after measuring and compensating the output of the system, the achieved response (the maximum dB SPL) was flat (73 ± 1 dB SPL) within the range of frequencies of interest (between 0.5 and 44 kHz). We have modified the paragraph (L.618-624) in order to clarify these details.

Methods, line 570-573: It would be interesting to show some tuning curves of the neurons recorded in mPFC, even though the authors state that the tuning seems to be very broad or unclear for pure tones. But in particular under these circumstances it would be important to understand how the individual stimuli were positioned within the tuning curves and how was the frequency of the Deviant stimulus selected?

Now we show examples of the frequency response areas (FRA) in Fig 2B. Note that the responses in the mPFC do no show clear receptive fields as in purely auditory structures, such as the AC; mPFC neurons seem to be more sensitive to certain sequences than to particular frequencies. Due to the “unstructured” nature of the receptive fields, it was not straightforward to choose a particular frequency for the deviant. Instead, we chose the 10 tones (gray dots in Fig. 2B) that would be used for the control sequences (aiming to include some tones that showed some amount of response, based both in the FRA and the manual search). Once the control sequences were recorded, we recorded as many oddball sequences as we could use each of those 10 tones as deviants (until we recorded all possible oddball sequences or the recording was not stable anymore).

These aspects of the experimental procedure are commented in Discussion (L.431-444) and Methods (L.676-705)

Methods, line 596: Two stimulus conditions were used for controlling for repetition suppression (RS): many standards and cascade. In order to control for properly for repetition, all stimuli of the control sequences should be within the receptive field of the neuron. Was that the case?

Please see the response to the previous comment and refer to Fig 2.

Methods, line 640-641: "We calculated the baseline spontaneous firing rate as the mean firing rate from 0-50 ms during the tone presentation." Why as the spontaneous firing rate computed during the tone presentation and not before the onset of the stimulation? 50 ms is a long time and some signals may have arrived in the PFC already by then.

Please see the response to your previous comment about Fig. 1C

Methods, line 707-709: post-hoc / observed "power analysis" was performed on the basis of the effect size iPE. I think that such a post-hoc power analysis does not yield relevant insights and therefore, it can be skipped (e.g. see Zhang et al. 2019:  Post hoc power analysis: is it an informative and meaningful analysis?).

The reviewer is right in the little relevance of post-hoc power analyses, so we have followed his/her suggestion and removed it.

Reviewer #4, Marta Garrido: This is a very exciting paper, done in a rigorous manner (with a number of controls), with data and findings that are quite compelling. I fully endorse the publication and only have minor comments that I hope the authors will find useful.

We would like to thank Dr. Garrido for her supportive comments and constructive criticism

1) Fig 1, C and D - is the recording site mPFC? Can this be specified please?

Yes, in the previous version both panels corresponded to recordings from mPFC. We have modified Fig. 1, including a schematic of the experiment in Fig. 1A. We hope it is clearer now.

2) There is a brief justification for why the cascade paradigm is a better control for repetition suppression (RS) than the many-standards. I thought this explanation wasn't clear enough (even the longer description in the methods was unclear) - can you say a bit more?

We have added a section dedicated to thoroughly explaining the control paradigms in Methods (L.626-673), trying to make clearer the differences between both control paradigms.

3) Fig 2 B, can the x labels be added? I assume this corresponds to freq but it's ambiguous.

We now show the measured indices within each plot, so they can be easily identified. These plots have been moved to Fig. 3B.

4) page 14, iPE>iMM, but isn't by definition according to 1B that iMM>=iPE?

Indeed. The neuronal indices mentioned in that page were calculated after baseline correction. Due to the long response durations and latencies, the baseline window could include portions of strong response, especially in the case in the control conditions (please see response to the editors). Using those measurements for response correction (aiming to remove the spontaneous activity) could in some cases result in “negative” corrected spike counts for the controls, which in turn would produce artifacts in the form of overestimated indices, such as the one noticed by the reviewer.

In the current analysis we do not apply baseline correction, in order to avoid this kind of artifacts.

5) In the Abstract, Introduction and Discussion the authors mention that it's unclear whether prediction errors are encoded independently of attention. There is actually a growing body of research in humans re modulations of prediction errors with attention, but that's not my point. The point I want to make is that "attention" is perhaps not the best concept to use here and instead "consciousness" would be more appropriate as this relates to the animals being under anaesthesia. There is a subtle but important distinction as even when the animal is conscious, it could still be inattentive. So these data show prediction errors without consciousness (and attention in this case) but these data could not answer the question of whether or not these prediction errors can be modulated by attention.

Dr. Garrido is correct, and this issue has been raised by another reviewer as well. We apologize for being overly speculative in this matter. Therefore, we have removed all references to attention from the manuscript and considered the suggestion about consciousness.

6) Fig 5, I thought this would flow better with the results section rather than discussion since this is still about data rather than a proposed model (which could be quite a nice add-on).

We thank you for the suggestion. We have included and expanded this data in a sub-section of Results (“Comparisons between the mPFC and the AC in the rat brain”, L.290) and moved the plot to Fig. 6, which is dedicated to compare the responses between mPFC and the auditory system.

7) In light of Fig 5, the authors propose that there is a progressive mitigation from the IC to AC and eventually vanishing at mPFC. However, I struggle to see that progressiveness - it appears to me more like an abrupt drop. Can this be tested statistically?

This is an excellent suggestion and we have compared the indices between AC from our previous work and the mPFC areas, finding that there are significant changes in both iPE and iRS but not in iMM, which supports a drastic (rather than progressive) change, as you have correctly noted (L.314-326).

8) As above but for iPE in the opposite direction- is it really a progressive build up? Again, can we test this statistically?

As mentioned before, the statistical comparison indicates sharp changes in iPE and iRS (L.314-326), which is a main finding in this paper.
