# Supplementary material for: Prediction error signaling explains neuronal mismatch responses in the medial prefrontal cortex
Source: PLoS Biol. 2020 Dec 21;18(12):e3001019. doi: 10.1371/journal.pbio.3001019 (PMC7785337; doi:10.1371/journal.pbio.3001019)
Supplement: Attachment — Submitted filename: response to reviewers.docx [file pbio.3001019.s007.docx]

----------------------------------------------------

Reviewer #2, Bernhard Englitz:

The authors have taken great care to improve the manuscript, and most of my points have been addressed.
There is one point, however, regarding the statistical analysis of the data, which still seems odd, and I could not see how the authors' response addressed this point.
While I agree that in relation to the DEV size (in relation to CTR), the difference between CTR and STD is small, but to the best of my (visual) assessment, should be significant, given the data shown in the previous Figure 4, now shown in Fig. 3D. Let's consider the right panel for region IL. If I understand correctly, the data in this panel are the same as the data in panel B (right), just displayed over time and differently normalized (see below). Is this correct?
If this is correct, then the distributions of the CTR and STD 'must' be different (of course I cannot assess the precise level of significance), but I think my visual estimate is accurate here.
In particular if the statistical analysis were to take the location inside the trial sequence into account (i.e. evaluating the difference CTR(iTrial) - STD(iTrial), there is a clear difference between them (which was more easily discernible in the previous figure 4 marker styles), since the dynamics over trials are contributing a large part of the variance. Depending on the number of conditions that went into the Friedman test, I could see how a multiple-testing correction reduced the significance again though.
While I think it is a relatively minor point, I think it is still important to clarify it. Maybe what is shown in Fig. 3B is still different than what is shown in Fig. 3D with respect to the statistical evaluation. You indicate that the data in Figure 3B is Median normalized, but I could not find in the manuscript, what this precisely meant: since the medians in Fig.3B are all different from 0 and 1, it can neither refer to separate divisions/subtractions of the median. Maybe it refers to the overall median, but even then I would expect the whole data to be centered around 0 or 1. Hence, the exact interpretation of this was not clear to me. If one of them is clearly preferable, maybe match the presentation in B and D?
For the benefit of the readers and to withstand all critical future examination I would ask the authors to clarify the procedure for the analysis here, and check in particular whether the STD responses are not infact slightly, but significantly smaller than the CTR responses.

Authors’ response:

We apologize to the reviewer for our explanations not being sufficiently clear to avoid further confusion and doubts regarding our data visualization choices. In short, the way in which data were displayed in the previous version of Fig 3B and D were not directly comparable. Whereas data in Fig 3B are normalized (as explained in *Data Analysis*, L733-747), data in Fig 3D were not. In the current version of Fig 3, both B and D use the same normalization method (as specified in L784-795), but there are still differences in the data processing workflow that prevent a direct comparison. We provide a more in-detail explanation in the following.

Regarding the previous version of Fig 3D using raw spike counts, we used all the 360 STD of the sequence, whereas to compute the responses to the STD (e.g., Fig 3B), we only used the 40 last-STD (i.e., occurring right before a DEV, as indicated in the Methods). It is essential to use all the STD trials in the time-course plots, otherwise the initial response decay occurring during the first trials would be lost.

In order to check to point raised by the reviewer, we looked for statistical differences between the spike counts of STD and CTR across the trial number. We computed the mean spike counts in groups of ten trials to obtain 40 measurements (to have the same number of data points for each condition), and then calculated the difference of these 40 spikes counts to STD minus the 40 spike counts to CTR. Because these distributions were not normally distributed we tested for statistical significance against zero with a Wilcoxon signed-rank test. All the fields but ACC demonstrated that across trial presentation mean spike counts for STD and CTR were significantly different (M2: p = 5.63 × 10^-08^, ACC: p = 0.22, PL: p = 5.91 × 10^-06^, IL: p = 2.45 × 10^-07^), as correctly noted by the reviewer. This result dovetails with the general finding of an iRS significantly greater than in the entire mPFC population, although quite small in size (median iRS = 0.06), as well as in M2 (when considering each area separately). However, as explained, the STD data included in this time course analyses using raw spike counts differed from those used to compute the mean STD responses anywhere else in the article where only the last-STD were considered.

To improve consistency across Fig 3, we have replotted Fig 3D normalizing the spike counts to the same Euclidean Norm vector corresponding to each unit that was previously calculated for the mean responses to the 40 DEV, the 40 CTR and the 40 last-STD (i.e., occurring right before a DEV, as in Fig 3B). Given that the Euclidean Norm vector was calculated for each unit based on the mean DEV, CTR and STD responses, some individual trials have values above 1, as can be seen in Fig 3D. Nevertheless, the overall shapes in the new version of Fig 3D do not differ much from the former version. We hope that this will be enough to avoid further confusion and to facilitate interpretation of Fig 3 to the reader.
